# Neutrophils Modulate Fibrogenesis in Chronic Pulmonary Diseases

**DOI:** 10.3389/fmed.2021.616200

**Published:** 2021-04-27

**Authors:** Lili Ding, Juan Yang, Chunmei Zhang, Xiuna Zhang, Pujun Gao

**Affiliations:** ^1^Department of Intensive Care Unit, The First Hospital of Jilin University, Changchun, China; ^2^Intensive Care Unit of Emergency Department, China-Japan Union Hospital of Jilin University, Changchun, China; ^3^Department of Hepatology and Gastroenterology, The Second Part of First Hospital, Jilin University, Changchun, China; ^4^Department of Hepatology, The First Hospital of Jilin University, Jilin University, Changchun, China

**Keywords:** neutrophils, asthma, chronic obstructive pulmonary disease, cystic fibrosis, idiopathic pulmonary fibrosis, fibrosis, fibrogenesis

## Abstract

Chronic inflammatory pulmonary diseases are characterized by recurrent and persistent inflammation of the airways, commonly associated with poor clinical outcomes. Although their etiologies vary tremendously, airway neutrophilia is a common feature of these diseases. Neutrophils, as vital regulators linking innate and adaptive immune systems, are a double-edged sword in the immune response of the lung involving mechanisms such as phagocytosis, degranulation, neutrophil extracellular trap formation, exosome secretion, release of cytokines and chemokines, and autophagy. Although neutrophils serve as strong defenders against extracellular pathogens, neutrophils and their components can trigger various cascades leading to inflammation and fibrogenesis. Here, we review current studies to elucidate the versatile roles of neutrophils in chronic pulmonary inflammatory diseases and describe the common pathogenesis of these diseases. This may provide new insights into therapeutic strategies for chronic lung diseases.

## Introduction

Pulmonary diseases are life-threatening conditions and an important cause of death worldwide. Chronic pulmonary diseases account for about 1 in 15 deaths in the United States, and mortality is increasing (1). According to current knowledge, chronic pulmonary diseases, including chronic obstructive pulmonary disease (COPD), asthma, cystic fibrosis (CF), and idiopathic pulmonary fibrosis (IPF), are characterized by chronic inflammation, repeated lung tissue injury and repair, and eventually pulmonary dysfunction. Although therapeutic strategies have been developed, effective therapy options are currently lacking for the middle and late stages of chronic pulmonary diseases (2). Fibrogenesis is a common phenomenon in the middle-to-late stages of these diseases, but the specific mechanisms are still unclear.

Generally, chronic inflammation may result from uncontrolled acute inflammation and may lead to fibrogenesis if repeated damage-repair cycles occur. Pulmonary fibrogenesis is a process of recurrent injury to the alveolar epithelium, followed by an uncontrolled proliferation of fibroblasts and overexpression of extracellular matrix (ECM) (3). Abnormal pneumocyte apoptosis, bronchiolar proliferation, and abnormal tissue remodeling are also involved in fibrogenesis (4). Fiber deposits in the alveolar interstitial matrix eventually leads to fibrosis. Neutrophils, a prominent subpopulation of immune cells in airways, have long been regarded as effector cells in the defense against extracellular pathogens during acute inflammation. However, neutrophils also participate in chronic inflammation in various diseases, such as rheumatoid arthritis (5, 6). Neutrophils play a modulating role in both innate and adaptive immune responses (7). On the one hand, neutrophils adhere to blood vessel walls, transmigrate to inflammatory sites following chemokine signals, and maintain cellular homeostasis by phagocytosis and degranulation or neutrophil extracellular trap (NET) formation (8). On the other hand, neutrophils and their effectors, including NETs, cytokines, exosomes, and autophagy, directly and indirectly activate fibroblasts, and promote ECM deposition. Most lung disorders, such as asthma, COPD, CF, and IPF, are inflammatory diseases that are accompanied by an increased number of neutrophils in the bronchoalveolar lavage fluid (BALF) or lung tissue (9–11). The long-term imbalance between pathogens and host defense contributes to the chronic inflammatory disease (12). High mobility group box protein 1 released by necrotic neutrophils induces inflammation and tissue remodeling by activating receptors for advanced glycation and toll-like receptor and CXC chemokine receptor (CXCR)4 signaling (13). As a result, chronic pulmonary interstitial inflammation, and fibrosis are induced and maintained.

In this review, we summarize how neutrophils restrict local inflammation and have a specific role in promoting chronic inflammation of the lung and pulmonary fibrosis. The review will reveal the various roles of neutrophils and explore common mechanisms of fibrogenesis in chronic lung diseases. This knowledge may inform treatment strategies for end-stage chronic lung diseases.

## Neutrophils as Defenders and as Inducers of Chronic Inflammation and Fibrosis of the Lung

Neutrophils contribute to nearly 60% of all leukocytes in human body fluids (14). More neutrophils are found in the pulmonary capillaries than in the systemic circulation, which facilitates their rapid entry into the lung tissues in response to infection and inflammatory stimuli (15). Primarily, they are recruited in initial phases to exert pro-inflammatory effects. These cells are recruited within minutes after injury and involved in the removal of tissue debris. Neutrophils possess a series of effector mechanisms to play their roles in disease. Apart from phagocytosis, the recently discovered NET formation, exosomes, release of cytokines and chemokines, and autophagy all play a vital role in pulmonary diseases. These protective mechanisms favor pathogen elimination and minimization of collateral damage (16), and they have been observed in multiple respiratory diseases, such as CF and asthma (17, 18). Following phagocytosis, neutrophils activate apoptotic pathways to limit the inflammation in the lung tissue.

Although neutrophils exert powerful antibacterial and antiseptic properties, studies have found that the neutrophil/total cell ratio in lung tissue is positively correlated with the lung fibrosis degree (19). This indicates that neutrophils and/or their products may contribute to fibrogenesis (20). The earliest pathological change of pulmonary fibrosis is the injury of pulmonary endothelial cells, followed by chronic injury of lung epithelial cells and deposition of lung interstitial matrix. Neutrophils induce endothelial and epithelial cell death directly through NET formation (21) or release of invading substance, such as angiopoietin-2, which promotes vessel destabilization and facilitates neutrophil influx to lung tissue (22). Neutrophils recruited to inflammatory sites affect the function of parenchymal cells or *via* their mediators, such as exosomes or cytokines (23, 24). Neutrophils secrete toxic mediators, such as reactive oxygen species (ROS) and reactive nitrogen species, that lead directly to tissue injury, or even worse, induce another wave of chemokines that forms a positive feedback loop. Moreover, some pathogens possess properties to subvert the immune system, which causes neutrophils to respond more aggressively, leading to untoward tissue injury (25). For example, neutrophil drive the inflammation-induced tissue damage in pulmonary tuberculosis and as such, neutrophil counts are correlated with chronic inflammation in tuberculosis (26).

### Phagocytosis and Secretion of Traditional Pro- and Anti-inflammatory Factors From Neutrophils in Chronic Lung Diseases

The cytoplasmic granules of neutrophils contain large amounts of lysozyme, alkaline phosphatase, antimicrobial proteins, defensin, and phagocytin. These substances eliminate foreign pathogens after their extracellular release by means of degranulation or after phagocytosis of the pathogen and fusion of the cytoplasmic granules to form the structure referred to as the phagolysosome (8). Phagolysosomes are formed from neutrophil granules (granule enzymes and ROS), lysosomes, and ingested bacteria (27). Pathogen killing is mediated by granule enzymes released from granules and ROS produced by mitochondria and NADPH oxidase (NOX) complexes. Mitochondria-generated ROS facilitate the innate immune function of neutrophils in a NOX-independent manner (28). By contrast, NOX is the only enzyme specifically used to produce ROS that participate in the regulation of infectious diseases and inflammation. However, NOX activity also leads to membrane depolarization of phagosomes to provide a proper environment for these cell organelles (29). The chemical elimination of pathogens prevents the further spread of infection and inflammation and protects the surrounding cells and tissues. Furthermore, proteinases and bactericidal proteins help in tissue repair and ECM reduction.

When phagocytosis is ineffective, degranulation occurs in response to stimuli. Neutrophils release granule contents in a degranulation manner. In this process, a series of enzymes, such as lysosomal enzymes and protease, are released. They help control bacterial challenge, but the ECM will be digested as a collateral side effect. Recurrent tissue damage and abnormal tissue repair contribute to lung tissue remodeling, degradation, and fibrogenesis. Neutrophil-derived matrix metalloproteinases (MMPs) digest the ECM, elastin, and collagen to allow for neutrophil transmigration from the bloodstream into tissues; but in some disease contexts, they weaken the host defense and contribute to tissue damage by degrading immune receptors and collectins (13). MMP2 and MMP9 are key orchestrators of emphysema (30, 31), indicating that MMPs are involved in pulmonary structural remodeling. In asthma, neutrophil phagocytosis is decreased (32), whereas, ROS generation is increased (33), which is highly correlated with tissue inflammation. In the COPD model, a reduction in MMP9 levels is correlated with disease alleviation (34). The dysregulated release of myeloperoxidase (MPO) and neutrophil elastase contributes to tissue damage and can exacerbate CF. Increased levels of MMP8/9 and neutrophil elastase are also detected in patients with CF (35). In the BALF and lung tissues from patients with IPF, high concentrations of neutrophil-derived MMP2 and MMP9 are detected, and MMP9 activity is positively correlated with the neutrophil count in the BALF (36). Collectively, the dysfunction of phagocytosis and degranulation favors ECM destruction, which is the basis for tissue over-repair, remodeling, and fibrogenesis.

### NETs – Double-Faced Newcomers in Chronic Lung Diseases

NETs are a mechanism of activated neutrophils to control infection and inflammation by “trapping” pathogens extracellularly in a suicidal or vital manner (37). The progress of active NET release is known as NETosis. NETs can be produced in response to infectious or non-infectious stimuli. Following activation of NET cell death programs, suicidal NETs are released by dying neutrophils 2–4 h after their activation (38) and are composed of decondensed chromatin (including cathelicidin LL-37, α-defensin, and neutrophil elastase), dissolved nuclear membranes, and chromatin. NETosis can be accelerated by the presence of bacteria as a way to wall off the infection and to limit the pro-inflammatory cytokine secretion, which limits the inflammatory response (39). Various agents, including bacteria, fungi, protozoa, viruses, platelets, interferon (IFN)-α, autoantibody, nitric oxide donors, can induce NET formation (40, 41). Moreover, granular enzymes and ROS positively regulate NET formation (41). *In vitro*, lipopolysaccharides can also induce the generation of NETs (42). These results indicate that NETs play a critical role in pathogen clearance and tissue homeostasis.

Connective tissue growth factor (CTGF, CCN2) is a matricellular protein implicated in fibrosis and an important downstream mediator of TGF-β induced fibrosis (43). The expression of CTGF is elevated in IPF, and confined to proliferating type II alveolar cell and myofibroblasts (44). CTGF may play a role in promoting fibroblast proliferation and extracellular matrix production in pulmonary fibrosis (44). Studies show a closely relationship between NET and CTGF. Myofibroblast CTGF expression is enhanced by NETs inducer (45), which also promote lung fibroblasts activation and myofibroblast differentiation (45, 46). Fibrotic interstitial lung biopsies demonstrate a close proximity between NETs and alpha-smooth muscle actin (α-SMA)-expressing fibroblasts (45).

NETs are reported to participate in the pathogenesis of multiple lung diseases, including pneumonia, acute lung injury, asthma, COPD, and CF. Although NETs play an important role in pathogen elimination and host defense, other evidence reveals substantial damage to the lung epithelium and endothelium, either directly or indirectly (21). Much of the literature on NETs in pneumonia, acute lung injury, COPD, asthma, and CF has already been reviewed in detail, so we will summarize major findings here (47). Recent data suggest that NETs also play an important role in autoimmune and autoinflammatory pathologies (48, 49). NETs induce the activation of lung fibroblasts, stimulate their differentiation into the myofibroblast phenotype, and promote fibrotic activity *via* the expression of interleukin (IL)-17, a primary initiator of inflammation and fibrosis (45). *In vitro*, isolated NETs significantly increase the levels of cytokines and stimulate macrophages to secrete IL-1β, which promotes neutrophil and macrophage infiltration in airways and contributes to lung fibrosis (50). As a result, NETs amplify tissue inflammation along the NET-cytokine-cell axis. Peptidylarginine deiminase 4 (PAD4), an essential enzyme for NET formation (51), is elevated in the lungs of COPD patients (52). In addition, the concentrations of NETs and their components, including extracellular DNA, LL-37, and CXCL8/IL-8, are increased in patients with neutrophilic asthma and COPD, and these elevated levels are negatively correlated with lung function (53). In COPD patients, NET levels are also elevated in the sputum, and they are similarly negatively correlated with lung function. In mice, reduced NET levels following erythromycin administration can alleviate emphysema and decrease the numbers of Th1, Th17, and myeloid dendritic cells (54). Furthermore, NET components stimulate histamine and leukotriene release, which may lead to further inflammatory changes in asthma and COPD. Neutrophils of CF patients exhibit a prolonged lifespan, which promotes NET production and increased necrosis (55). Furthermore, the neutrophil count is increased in patients with CF, and the levels of NET components (extracellular DNA, neutrophil elastase, and MPO) are correlated with disease severity (56). In addition, increased levels of autoantibodies against NET components are correlated with decreased lung function in patients with CF (56, 57). Despite no currently proven direct connection between NETs and IPF, the relationship between NET and pulmonary fibrosis formation suggests a likely relationship between IPF and NETs. A recent study showed that pulmonary surfactant protein-D can inhibit NETosis in lipopolysaccharide-stimulated human neutrophils, providing the potential to eliminate negative NET effects (58).

### Exosomes – Tiny Multi-Agent Messengers in Chronic Lung Diseases

Exosomes are extracellular vesicles <150 nm in size that are released into the plasma by budding and carry proteins, lipids, and nucleic acids. Exosomes are secreted by a wide range of cells in response to different stimuli and play a role in information transmission (59). As an intercellular communicator, exosomes can reach distant cells quickly and affect their behavior *via* interaction with surface markers. Exosomes acquiring the CD63/CD66b phenotype are from neutrophils, with which they can be traced extracellularly. Studies have shown that exosomes are released into the lungs, and they are related to the pathophysiology of lung diseases (60). Functionally, exosomes eliminate pro-inflammatory chromosomal DNA fragments to maintain cellular homeostasis (61). Functional inhibition of exosomes triggers the innate immune response to generate more ROS by accumulating nuclear DNA.

Exosomes from activated neutrophils express higher neutrophil enzyme than quiescent exosomes, which indicate that exosomes show higher destructive effect in lung (62). Neutrophil-derived exosomes can be immediately internalized by airway smooth muscle cells, thereby altering their proliferative properties and contributing to airway remodeling (17). This is associated with excessive neutrophil-driven inflammation and local elevation in neutrophil elastase levels. Exosomes from neutrophils enhance the proliferation of airway smooth muscle cells to promote airway remodeling in patients with asthma, and they play an important role in the progression of asthma (17). In COPD, exosomes containing neutrophil elastase, which is resistant to alpha1-antitrypsin, destroy the tissue architecture *via* integrin Mac-1 and neutrophil elastase (62). Although studies concerning neutrophil-derived exosomes and lung fibrotic disease have not been identified, such exosomes potentially contribute to the pulmonary architectural distortion in chronic lung diseases. In turn, exosomes from lung fibroblasts activate DNA damage response and epithelial cell senescence (63). In systemic sclerosis, a systemic fibrotic disease, neutrophil-derived exosomes inhibit the proliferation, and migration of human dermal microvascular endothelial cells *via* S100A8/A9 in neutrophils, this result indicates that exosomes from neutrophils prevent lung remodeling (64). In mice with bleomycin-induced pulmonary fibrosis, exosomes containing microRNA-22 can ameliorate pulmonary fibrosis by suppressing transforming growth factor (TGF)-β1-induced expression of α-smooth muscle actin (65). This contrary effect of exosomes in fibric diseases is mainly caused by the different functional components contained in the released exosomes.

### Autophagy, an Unusual Cell Death in Chronic Lung Diseases

Autophagy is the lysosome-dependent process of cell organelle self-degradation that is mediated under various cellular stress conditions to maintain cellular homeostasis of cellular protein synthesis, degradation, and recycling (66). Autophagy is crucial for cellular function and can be seen in both physiological and pathological processes in most cells, including neutrophils. In recent studies, autophagy has shown a complicated and important moderator function in pulmonary diseases (67). In chronic lung diseases, autophagy helps to maintain the cell cycle of lung fibroblasts and reduce the production of ECM.

In patients with asthma, autophagy levels of peripheral blood cells in the sputum and peripheral blood eosinophils are increased (68). Similarly, autophagy levels in airway tissues are also increased in a murine asthma model. Inhibiting autophagy reduces airway responsiveness, eosinophilia, and inflammation in this murine asthma model (69). Smoke-exposed neutrophils present increased autophagy and lose the ability to ingest the respiratory pathogen *Staphylococcus aureus* (70). Moreover, cigarette smoke exposure induces autophagic cell death in neutrophils, leading to the development of emphysema (71). These results indicate that the upregulation of autophagy in neutrophils contributes to persistent inflammation and the development of COPD.

However, studies concerning the autophagy of neutrophils in CF and IPF are lacking. The level of autophagic Beclin-1 is decreased in fibroblasts in fibrotic autoimmune diseases, suggesting an inhibited autophagy status (72). Autophagy stimulated by rapamycin (an mTOR inhibitor promoting autophagy) causes the reduced expression of fibronectin and α-smooth muscle actin in fibroblasts *in vitro* and exerts an anti-fibrotic effect in the bleomycin model *in vivo* (73). By contrast, inhibiting autophagy leads to an increase in extracellular collagen production and fibroblast-myofibroblast differentiation in pulmonary fibrosis (74, 75). In IPF, insufficient autophagy promotes fibrogenesis by stimulating fibroblast proliferation (76) and accelerates cellular senescence and myofibroblast differentiation of lung fibroblasts, suggesting the pro-fibrotic functions of insufficient autophagy in IPF (77, 78). Macroautophagy and mitophagy are decreased in lung epithelial cells and lung fibroblasts of IPF patients (54). Mitophagy, a crucial process in cellular energy homeostasis, modulates macrophage apoptosis and stabilizes macrophages to release TGF-β1, thereby stimulating local fibroblast activation. Aging mitochondria and impaired autophagy/mitophagy are involved in the pathogenesis of IPF (75, 79). This result caters to the fact that aging is a major risk factor for IPF (80). Blocking mitophagy in alveolar macrophages protects against bleomycin-induced fibrosis in mice (81). These results indicate an intimate connection between autophagy and persistent inflammation, as well as fibrosis, but more studies are needed to verify the specific function of neutrophil autophagy in these diseases.

Some drugs show to be highly associated with autophagy. For example, hydroxychloroquine blocks autophagy by impairing the autophagosome-lysosome fusion and the degradation of the autophagosome contents (82). Metformin, an insulin-sensitizing drug, stimulates AMP-activated protein kinase, which enhances autophagy by ULK1 (a key regulating protein of autophagy) activation (83). Moreover, autophagy positively regulates NET formation (84, 85), proposing that inhibition of autophagy using pharmacological inhibitors may hinder the release of NETs. Consequently, modulation of autophagy emerges as a promising therapeutic option for the treatment of NET-driven disorders.

### Cytokines and Chemokines as Positive and Negative Regulators in Chronic Lung Diseases

Neutrophils secrete a vast number of cytokines and chemokines, such as pro-inflammatory cytokines (IL-1, IL-6, IL-17, and IL-18), anti-inflammatory cytokines (IL-10, TGF-β1, and TGF-β2), chemokines (CXCL1–11, CCL2–4, and CCL17–22), and immunoregulatory cytokines IFN-γ, IL-12, and IL-23), all of which act directly or indirectly on other immune cells, contributing to tissue damage or repair (8, 29, 86). As a key connector of the innate and adaptive immune system, neutrophils interact with other immune cells, including T cells, B cells, natural killer cells, macrophages, dendritic cells, and mesenchymal stem cells (7). Particularly, IFN-γ, TGF-β, IL-6, IL-17A, and IL-17F are functionally important in lung diseases.

IFN-γ shows anti-fibrotic effects by inhibiting the collagen formation in fibroblasts *in vitro*. It also decreases the gene expression of pro-fibrogenic factors, including TGF-β1 and CTGF (87). Clinically, INF-γ is used to treat lung fibrosis in IPF, despite its effectiveness being quite controversial (88).

TGF-β is a strong pro-fibrotic factor in the lung (89). Three major mammalian isoforms of TGF-β have been identified: TGF-β1, TGF-β2, and TGF-β3. TGF-β1 and TGF-β2 are secreted by neutrophils. TGF-β induces the proliferation of fibroblasts and macrophages *via* platelet-derived growth factor expression and stimulates fibroblasts to generate superabundant ECM. Besides, activated macrophages also express a vast number of pro-inflammatory and fibrogenic cytokines, such as tumor necrosis factor-α, IL-1β, and IL-13, triggering persistent inflammation downstream and chronic lung fibrosis (89). Pulmonary TGF-β1 levels are increased in a model of experimental lung fibrosis, and TGF-β1 overexpression induces persistent pulmonary fibrosis *via* the SMAD3 signaling pathway (90). *Smad3* gene knockout protects mice from TGF-β1- and bleomycin-induced pulmonary fibrosis (91).

In patients with IPF or acute exacerbation of IPF, IL-6 levels in the BALF and serum are significantly higher compared to healthy controls (92–94), indicating a close connection between this cytokine and fibrosis pathogenesis. In IPF, IL-6 enhances the proliferation of lung fibroblasts *via* SHP-2/ERK/MAPK signaling (95). In addition, IL-6 promotes in IPF the resistance of lung fibroblasts to Fas-induced apoptosis by overexpression of the anti-apoptotic protein BCL-2 (96).

In human lung fibroblasts, IL-17A stimulates their proliferation, generation of ECM, and differentiation into the myofibroblast phenotype *via* the NF-κB pathway. IL-17 also promotes fibrosis *via* NETosis (97). IL-17 expresses in NETs and promotes the fibrotic activity of lung fibroblasts (45). Study shows that IL-17-producing cells and NETosis are synchronous increased in psoriatic lesions. The expression of IL-17 is increased in presence of NETs *in vitro* (97). Neutralization of IL-17A can ameliorate bleomycin-induced lung fibrosis in mice (98). These results indicate a pro-fibrotic role for IL-17A in human lung tissue remodeling through direct effects on lung fibroblasts (99). Superabundant neutrophils also lead to tissue damage, and pulmonary fibrosis is significantly alleviated when neutrophils are depleted (100). Collectively, neutrophils, as a major source of inflammatory factors, play vital pro- or anti-fibrotic roles in the lung. This dual effect is mainly caused by differences in environmental conditions of the distinct pulmonary diseases. Changing disease-related factors may reverse the disease progression and promote recovery.

## Summary

**Figure 1:**
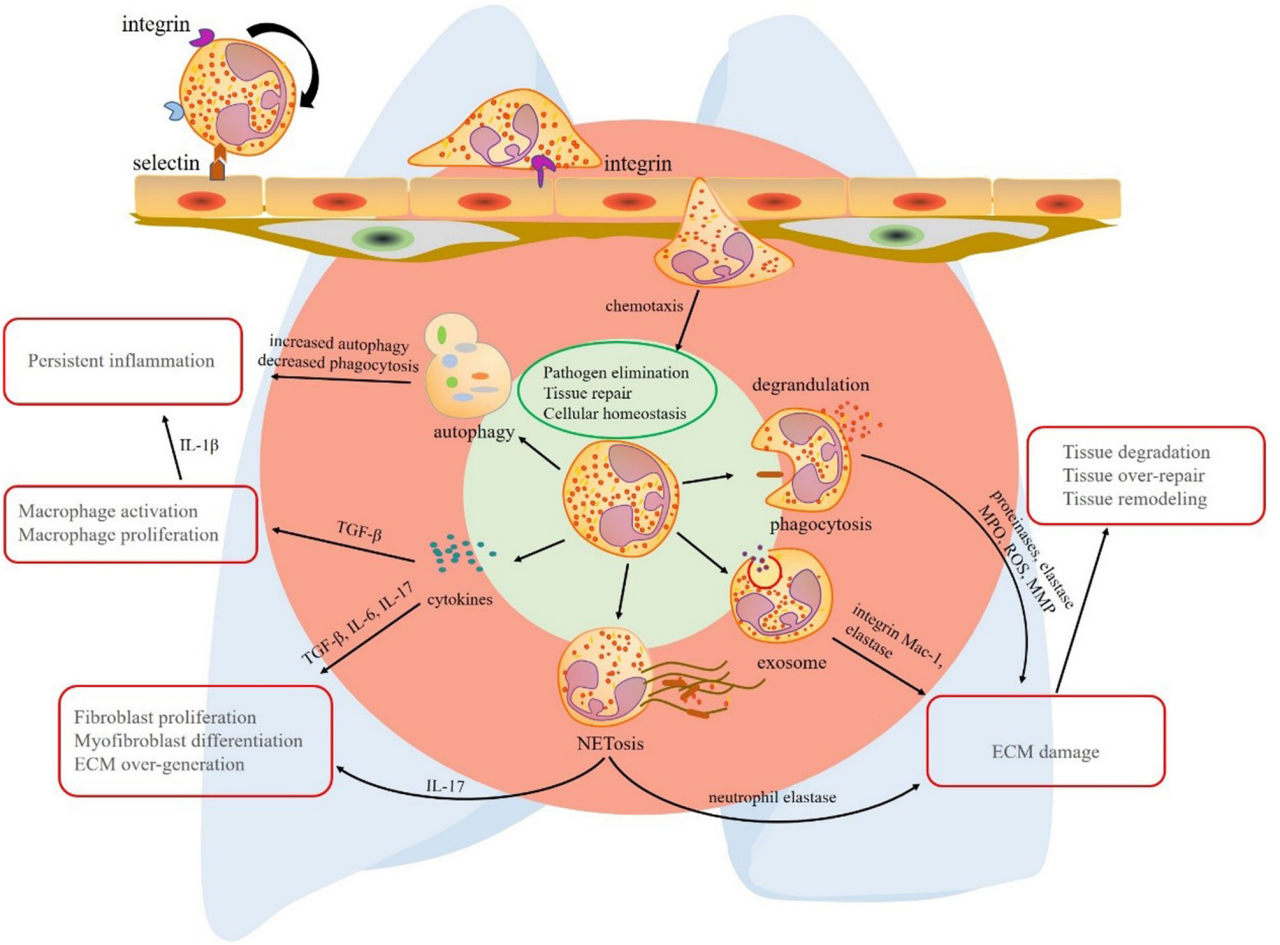
The multiple effects of neutrophils in lung fibrosis. Circulating neutrophils roll, adhere, crawl, and transmigrate to chemokines to lung tissue. In the lung tissue, neutrophils play their roles *via* phagocytosis, degranulation, neutrophil extracellular trap (NET) formation, exosome secretion, release of cytokines, and autophagy redox balance. The primary common features of these mechanisms are pathogen elimination, tissue repair, and cellular homeostasis, respectively. However, the mechanisms that promote the progression of chronic inflammatory lung disease are quite different. Phagocytosis, degrandulation, NET formation, and exosomes contribute to ECM injury and tissue damage-repair-remodeling. In addition to ECM damage, NETs play a role in activating immune responses and release pro-fibrotic factor IL-17. Although neutrophil autophagy effect is limited, increased proportion of autophagy in neutrophil deduces the ability of eliminating inflammation. A vast of pro-fibrotic cytokines released by neutrophils contribute to lung fibrosis formation, respectively. Transforming growth factor (TGF)-β promotes persistent inflammation by macrophage activation and proliferation. interleukin (IL)-17 derived from neutrophils or NETs induces fibroblast proliferation and myofibroblast differentiation. As a result, extracellular matrix (ECM) is over-expressed in interstitial tissue of lung. TGF-β, transforming growth factor-β; IL-17, interleukin-17; NET, neutrophil extracellular trap; ECM, extracellular matrix; MPO, myeloperoxidase; ROS, reactive oxygen species; MMP, matrix metalloproteinase.

Chronic inflammatory lung diseases are a group of neutrophil-related disorders with poor prognosis in middle-to-late stages. To sum up the common features of these diseases, first, neutrophilia can be detected in the lung tissue or BALF. Second, protective factors and pro-inflammatory factors coexist, but the balance is disturbed under disease conditions. Third, specific neutrophil functions are altered, such as enhanced ROS production, aberrant NET formation, increased autophagy, and abnormal secretion of cytokines. In contrast to the traditional view on these short-lived cells, research corroborates the hypothesis that neutrophils and their products contribute to inflammation removal, but also chronic inflammation and fibrosis of lung tissue (Figure 1). The shift in the balance toward tissue destruction may result in persistent inflammation and fibrogenesis. These mechanisms also explain the acute exacerbation of some chronic lung diseases after experiencing infection. Avoiding infection is an important preventive measure to control pulmonary fibrosis.

Given the role of neutrophils in fibrosis, strategies focusing on neutrophil components may be effective, such as reducing neutrophil numbers in the airway, decreasing protease and ROS generation, decorating NETs, regulating autophagy, reducing the expression or activity of TGF-β protein, and providing exogenously exosomes containing microRNA. DNase and NET-associated elastase that affect the formation of NET may be helpful. Compound, such as hydroxychloroquine, which can reduce NETs *via* blocking autophagy is also considerable. Moreover, based on the tightly correlation of NETs and lung function, NET is expected to be a biomarker for evaluating criteria of lung function and fibrosis. However, the effectiveness of these strategies is limited by their off-target effects. More studies are needed to explore the precise role of neutrophils in lung fibrotic diseases, which will provide better evidence for the treatment of these diseases.

## Author Contributions

LD wrote this review. JY and CZ consulted relevant literature. XZ drew the figures. PG revised the manuscript. All authors contributed to the article and approved the submitted version.

## Conflict of Interest

The authors declare that the research was conducted in the absence of any commercial or financial relationships that could be construed as a potential conflict of interest.
